# Targeting conserved epitopes in structural proteins: a next-generation vaccine strategy against the newly identified HKU5-CoV-2 virus

**DOI:** 10.1038/s41392-025-02257-0

**Published:** 2025-05-21

**Authors:** Leonardo Pereira de Araújo, Evandro Neves Silva, André Luiz Caliari Costa, Patrícia Paiva Corsetti, Leonardo Augusto de Almeida

**Affiliations:** Laboratory of Molecular Biology of Microorganisms, Federal University of Alfenas (UNIFAL), Alfenas, 37130-001 Minas Gerais, Brazil

**Keywords:** Infectious diseases, Vaccines

A recent study published in *Cell* by Chen et al., highlighted by *Nature* journal, identified the *HKU5-CoV-2* lineage, a coronavirus detected in bats with the potential to interact with the human ACE2 receptor.^1,2^ Experiments conducted in intestinal and airway cells indicated that *HKU5-CoV-2* binds to the receptor less efficiently than *SARS-CoV-2*. However, the broad ACE2 ortholog tropism suggests a potential risk of adaptation to the human host.^1,2^

The spillover of zoonotic viruses poses a persistent challenge to global health, driving successive health crises.^3^ The COVID-19 pandemic, caused by *SARS-CoV-2*, resulted in approximately 7 million deaths, but it was not an isolated event.^4^ Recurring outbreaks of emerging and re-emerging viruses, such as *SARS-CoV*, *MERS-CoV*, and Mpox, among others, highlight a concerning epidemiological pattern.^4^ These events underscore the need for continuous surveillance, genomic monitoring, and robust preventive strategies to anticipate and mitigate future pandemic threats.^3,4^

Given this emerging threat, antiviral strategies such as neutralizing antibodies, fusion inhibitors, and small molecules—including nirmatrelvir, remdesivir, and GC376—have been evaluated and demonstrated potential against *HKU5-CoV-2*.^1,2^ However, the absence of diagnostic and vaccine tools exposes our vulnerability to a potential “Disease X“.^1^ The infectivity of this virus has not yet been assessed in vivo, making further investigations essential.^1^ For this reason, we intend to determine whether epitopes previously identified by our research group in *SARS-CoV-2* structural proteins are conserved in *HKU5-CoV-2*, following an approach validated by our team in a study previously published in *The Lancet Microbe* in 2024 for the Mpox and *Alaskapox* viruses.^4,5^ If conservation was not confirmed, we aimed to identify new epitopes in the structural proteins of this virus with potential applications in diagnostics and as vaccine candidates.

The sequences of the structural proteins—envelope protein, membrane glycoprotein, nucleocapsid phosphoprotein, and spike glycoprotein—were obtained from the GenBase database (accession numbers: C_AA085189.1, C_AA085190.1, C_AA085191.1, C_AA085192.1, C_AA085193.1, and C_AA085194.1). We then aligned previously identified *SARS-CoV-2* epitopes with *HKU5-CoV-2* proteins. Additionally, we performed predictions of B-cell and T-cell (MHCI and MHCII) epitopes and analyses related to antigenicity, allergenicity, physicochemical properties, transmembrane regions, N-glycosylation sites, and three-dimensional modeling of the spike glycoprotein, following the methodology previously described by our group.^4,5^

**Fig. 1:**
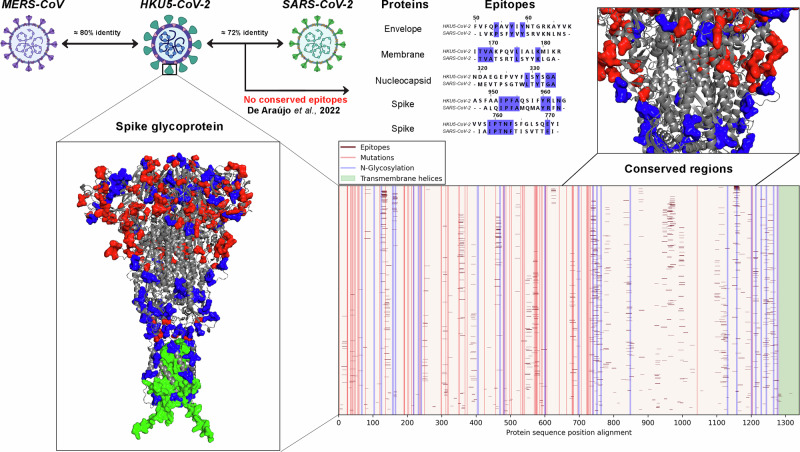
Comparison of *MERS-CoV, HKU5-CoV-2*, and *SARS-CoV-2* proteins, emphasizing amino acid identity and differences in the Spike glycoprotein. The top diagram illustrates the phylogenetic relationship between these viruses and the lack of conserved epitopes between *HKU5-CoV-2* and *SARS-CoV-2*. The three-dimensional structure of Spike, predicted using AlphaFold3, and the accompanying graph highlight N-glycosylation sites (blue), mutations (red), and transmembrane regions (green). The lower graph presents the protein sequence alignment, marking epitopes in brown and conserved regions, offering a comprehensive view of structural and functional variations

Given the approach previously reported for Mpox and *Alaskapox*, we investigated the conservation of *SARS-CoV-2* epitopes in *HKU5-CoV-2*, which could enable a bivalent vaccine candidate.^5^ However, our findings reveal that *HKU5-CoV-2* shares only 80% amino acid sequence identity with MERS-CoV and 72% with *SARS-CoV-2*, indicating significant structural differences. This divergence may explain the lack of conservation of previously mapped epitopes, as described in Fig. 1. Furthermore, in the analysis of the six available *HKU5-CoV-2* genomes, we identified 77 mutations in the spike protein, further reinforcing the substantial structural alterations of this virus. As represented in Fig. 1, most of these mutations (shown in red in the three-dimensional structure) and N-glycosylations (shown in blue) are located in the RBD region, while the central region exhibits a higher degree of conservation, represented in gray.

Despite these mutations, the *HKU5-CoV-2* spike glycoprotein exhibited approximately 1088 antigenic and stable epitopes, along with a high number of N-glycosylation sites. These characteristics, together with the identified mutations, reflect a pattern similar to that observed by our research group in *SARS-CoV-2*.^4^ Nonetheless, we identified a conserved region between amino acids 750 and 1200, with only one mutation at residue 1043 (Fig. 1). In addition to spike, structural proteins such as envelope, membrane, and nucleocapsid also exhibited conserved and antigenic epitopes (data not shown), highlighting these potential targets for vaccine development and the need for continuous monitoring of this virus.

The identified conserved region emerges as a promising target for the development of effective vaccines. Furthermore, as demonstrated by our group and suggested by Chen et al.,^1,5^ conserved epitopes across different viral species can be explored to create broad-spectrum immunological strategies. Identifying mutated epitopes not only enables tracking of viral evolution but also facilitates the development of immunodiagnostic tools for the differential early detection of emerging variants. Nonetheless, it is important to consider that, despite their high conservation, the epitopes may exhibit suboptimal antigenicity, potentially limiting their ability to induce potent neutralizing antibody responses. This highlights the essential role of subsequent in vitro and in vivo studies to validate their immunogenic potential and functional relevance in protective immunity.

The development of immunodiagnostic tests and vaccines against *HKU5-CoV-2* is essential. Our findings confirm that its structural proteins exhibit antigenicity patterns similar to those of *SARS-CoV-2*. Thus, as highlighted by Chen et al. and corroborated by our investigations, the conservation of epitopes in zoonotic viruses should be extensively explored to strengthen our defenses against future pandemics.
